# The role of serine/threonine phosphatases in human development: Evidence from congenital disorders

**DOI:** 10.3389/fcell.2022.1030119

**Published:** 2022-10-13

**Authors:** Pieter Vaneynde, Iris Verbinnen, Veerle Janssens

**Affiliations:** ^1^ Laboratory of Protein Phosphorylation and Proteomics, Department of Cellular and Molecular Medicine, University of Leuven (KU Leuven), Leuven, Belgium; ^2^ Leuven Brain Institute (LBI), Leuven, Belgium

**Keywords:** serine/threonine protein phosphatase, *de novo* mutation, congenital disease, neurodevelopmental disorders, sex development disorder

## Abstract

Reversible protein phosphorylation is a fundamental regulation mechanism in eukaryotic cell and organismal physiology, and in human health and disease. Until recently, and unlike protein kinases, mutations in serine/threonine protein phosphatases (PSP) had not been commonly associated with disorders of human development. Here, we have summarized the current knowledge on congenital diseases caused by mutations, inherited or *de novo*, in one of 38 human PSP genes, encoding a monomeric phosphatase or a catalytic subunit of a multimeric phosphatase. In addition, we highlight similar pathogenic mutations in genes encoding a specific regulatory subunit of a multimeric PSP. Overall, we describe 19 affected genes, and find that most pathogenic variants are loss-of-function, with just a few examples of gain-of-function alterations. Moreover, despite their widespread tissue expression, the large majority of congenital PSP disorders are characterised by brain-specific abnormalities, suggesting a generalized, major role for PSPs in brain development and function. However, even if the pathogenic mechanisms are relatively well understood for a small number of PSP disorders, this knowledge is still incomplete for most of them, and the further identification of downstream targets and effectors of the affected PSPs is eagerly awaited through studies in appropriate *in vitro* and *in vivo* disease models. Such lacking studies could elucidate the exact mechanisms through which these diseases act, and possibly open up new therapeutic avenues.

## 1 Introduction

Reversible protein phosphorylation lies at the core of protein regulation, fine-tuning their activity and stability in cells and tissues. Through addition or removal of a negatively charged phosphate group, protein activity can be up- or downregulated, or their stability promoted or inhibited. In homeostatic conditions, a delicate phosphoproteomic balance is achieved through the counterbalanced action of phosphorylating kinases, and dephosphorylating phosphatases. Consequently, when this balance is disrupted, disease inevitably follows, making kinases and phosphatases prime therapeutic targets in cancer, neurodegenerative diseases, diabetes, cardiac diseases, immune dysfunction, development, and other human pathologies. As protein kinases were discovered several decades ahead of protein phosphatases, the latter enzymes generally lag a bit behind their enzymatic antagonists, both in terms of general understanding of their function and regulation, and in terms of their implication or therapeutic targeting in human disease (Brautigan, 2013; Tonks, 2013).

Regulatory phosphorylation occurs on either a serine, threonine, or tyrosine residue. Due to tyrosine having a benzene ring, and the similar structure between serine and threonine, two major classes of protein phosphatases have been identified, the serine/threonine phosphatases (PSP), and the protein tyrosine phosphatases (PTP). In this review, we will focus on the serine/threonine phosphatase family of proteins, highlighting their roles in inborn genetic disorders.

## 2 Structure and regulation of Ser/Thr phosphatases

**FIGURE 1:**
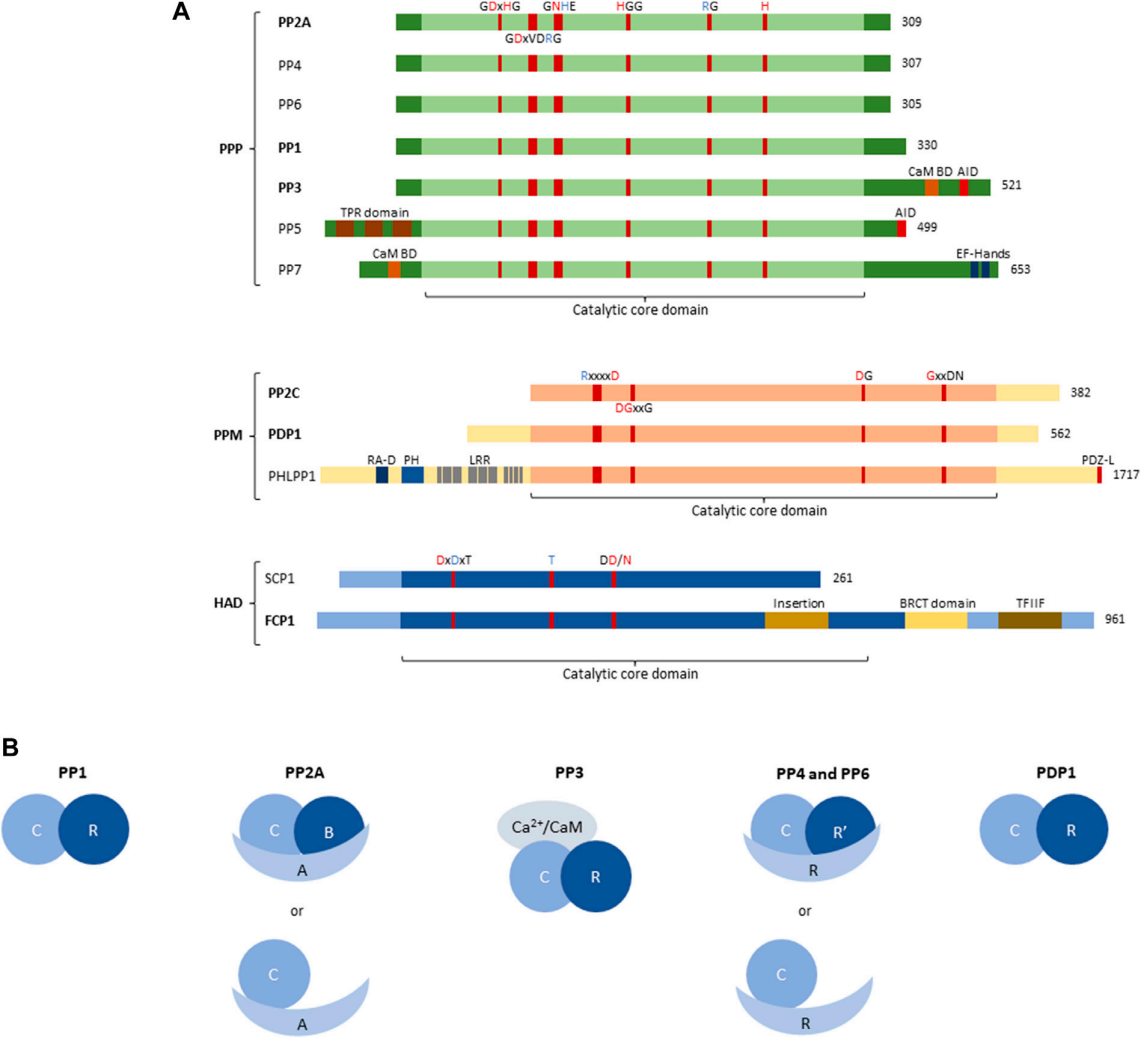
Structure of Protein Serine/Threonine phosphatase (PSP) superfamily. **(A)** Structural and domain organisation of the catalytic subunits of the main PSP phosphatases. PSPs can be subdivided into three subfamilies, the Phosphoprotein phosphatases (PPP), the Metal-dependent phosphatases (PPM), and the Haloacid phosphatases (HAD). The central part of each PSP phosphatase denotes the catalytic core domain, with relevant conserved amino acids and their position annotated. A red amino acid denotes involvement in metal binding, blue denotes phosphate binding. Additional structural and regulatory domains are indicated as follows: CaM BD, Ca^2+^-calmodulin binding domain; AID, auto-inhibitory domain; TPR domain, tetratricopeptide domain; EF-hands, canonical Ca^2+^-binding motif; RA-D, Ras-association domain; PH, Pleckstrin homology domain; LRR, leucine-rich repeat; PDZ-L, PDZ Ligand; BRCT domain, BRCA1-related C-terminal domain; TFIIF, transcription factor interacting helix. Bold indicates involvement in inborn genetic disease. **(B)** Organisation of PSP holoenzymes. PP1 forms a dimer between the catalytic subunit (3 isoforms), and regulatory PIPs (∼180). PP2A predominantly forms trimers between the catalytic C-subunit (2 isoforms), the structural A-subunit (2 isoforms), and the regulatory B-subunit (>25 isoforms); however, dimers between the catalytic and structural subunit have also been described. PP3/calcineurin/PP2B forms trimers consisting of the catalytic subunit (3 isoforms), a Ca^2+^-binding regulatory subunit (2 isoforms) and Ca^2+^-calmodulin. PP4 and PP6, like PP2A, can form both dimers and trimers, with trimers consisting of the catalytic subunit (1 isoform each), a structural subunit (R, 3 isoforms each) and a regulatory subunit (R′, 2 and 3 isoforms, respectively), and dimers consisting of the catalytic and a structural subunit. PDP1 is the only non-PPP phosphatase that forms a holoenzyme with one regulatory subunit (R, 1 isoform). C, catalytic subunit; R, scaffold or regulatory subunit; R′, regulatory subunit; A, scaffold subunit of PP2A; B, regulatory subunit of PP2A; CaM, calmodulin.

PSP phosphatases specifically dephosphorylate phospho-Ser or phospho-Thr residues, which, throughout the proteome, represent the large majority of protein phosphorylation, with phospho-serine estimated 86.4% and phospho-threonine 11.8% (Olsen et al., 2006). In humans, PSP phosphatases consist of three distinct subfamilies, the phosphoprotein phosphatases (PPP), the metal-dependent protein phosphatases (PPM), and the Asp-based HaloAcid Dehalogenase (HAD) phosphatases – which largely differ from each other in their specific structures and catalytic mechanisms (Figure 1) (Shi, 2009; Janssens, 2021).

### 2.1 Phosphoprotein phosphatases

The subfamily of phosphoprotein phosphatases (PPP) are further subdivided into seven different classes, PP1 through PP7 (Figure 1A), all of which, but PP5 and PP7, form holoenzymes (Figure 1B).

The identification and characterization of PPPs took a flight in the 1990s, using novel genetic and molecular tools. Then, it was noticed that certain, sometimes unidentified, phosphatases contained several conserved elements, and could thus be grouped within the same family (Cohen, 1997). PPPs are characterised by several conserved amino acid sequences in their catalytic subunits, with certain residues responsible for metal binding, and others responsible for phosphate binding (Figure 1A) (Shi, 2009). The coordinated metal ions bind and activate a water molecule that performs the nucleophilic attack on the P-atom of the phosphate group to remove it from the substrate (Shi, 2009). In addition to the relatively conserved catalytic core domain, which entails the highly conserved metal- and phosphate-binding residues, other regulatory domains can be present (Figure 1A). PP3 (also called PP2B or calcineurin), for example, has an additional C-terminal extension, containing a calmodulin-binding domain, and an auto-inhibitory domain. The latter domain constitutively represses PP3 activity, but upon binding of Ca^2+^/calmodulin, PP3 becomes active (Roy and Cyert, 2020). PP5 also contains an auto-inhibitory domain located in the C-terminal extension, which holds the N-terminal extension in place to physically block the catalytic core domain. Upon binding of the tetratricopeptide domain within the N-terminal extension to a substrate, this inhibition gets lifted to allow dephosphorylation (Golden et al., 2008). PP7, also acting as a monomer, contains a calmodulin-binding domain as well, located in the N-terminal extension. Furthermore, in the C-terminal extension of PP7, EF-hands are present, providing an extra regulation of activity through Ca^2+^ (Shi, 2009).

The remaining PPPs – PP1, PP2A, PP4, and PP6 – lack any regulatory domains and rely on interaction with regulatory subunits for targeting and regulation of activity (Janssens, 2021) (Figure 1B). In addition, PP3 also forms a complex with one regulatory, Ca^2+^-binding subunit, despite having two regulatory domains itself (Figure 1B). PP1 binds a wide variety of regulatory subunits, called PP1-interacting proteins (PIPs), of which more than 180 are known and which act as targeting subunits, substrate specifiers, substrates, or inhibitors (Figure 1B). They combine short linear motifs (*e.g*. RVxF, SILK, MyPhoNE,…) to form large and unique interaction interfaces with PP1 (Verbinnen et al., 2017). Additionally, PP1 has been described to form trimers as well; however, this is less common than dimer formation (Verbinnen et al., 2017). PP2A on the other hand, mainly forms trimeric holoenzymes consisting of the catalytic C-subunit, a regulatory B-subunit and a scaffolding A-subunit (Figure 1B) (Janssens and Goris, 2001). Here, two isoforms of both the C- and the A-subunit are known, while 15 B-subunit encoding genes express over 25 B-subunit isoforms through alternative splicing/translation initiation, forming more than 100 trimers (Lambrecht et al., 2013). Furthermore, dimers of PP2A between the C- and A-subunit have been described, although to a lesser extent than trimers (Janssens and Goris, 2001). The B-subunits define substrate specificity of PP2A, in part by the presence of specific Short Linear Interaction Motifs (SLIMs) that function as substrate docking motifs (Cundell et al., 2016; Hertz et al., 2016; Kruse et al., 2020; Fowle et al., 2021). The PP2A-related phosphatases PP4 and PP6 also form trimers and dimers, alike to PP2A (Figure 1B), but the overall number of holoenzyme complexes is significantly smaller (Ohama, 2019; Park and Lee, 2020).

### 2.2 Metal-dependent phosphatases

A second subfamily of PSPs are the PPMs, being magnesium/manganese ion-dependent in their phosphatase activity. The PPM subfamily can be further subdivided into the ‘classical’ PPMs, also called type 2C protein phosphatases (PP2C), the pleckstrin homology domain leucine-rich repeat protein phosphatases (PHLPP), and the pyruvate dehydrogenase phosphatases (PDPs). All these phosphatases share a conserved catalytic core region, with highly conserved amino acid sequences responsible for phosphate- or metal-binding (Figure 1A) (Shi, 2009). The PP2C family has thirteen members (*PPM1A, PPM1B, PPM1D, PPM1E, PPM1F, PPM1G, PPM1H, PPM1J, PPM1K, PPM1L, PPM1M, PPM1N*, and *PPM1O*, also known as *ILKAP*) which are highly similar but differ in substrate specificity and activity (Kamada et al., 2020). The PHLPPs and PDPs each consist of two members, *PHLPP1* and *PHLPP2*, and *PDP1* and *PDP2*. As the only member of the PPM superfamily to form holoenzymes, PDP1 forms a dimer with a regulatory subunit (Figure 1B) (Janssens, 2021). PHLPPs contain several regulatory domains within the protein structure, such as the pleckstrin homology domain, the RAS-association domain, and leucine-rich repeats in the N-terminal extension, and the PDZ-ligand in the C-terminal extension (Figure 1A) (Baffi et al., 2021).

### 2.3 Haloacid dehalogenase phosphatases

The third PSP subfamily of HAD phosphatases contains seven members (*CTDP1, CTDSP1*, *CTDSP2*, *CTDSPL*, *CTDSPL2*, *CTDNEP1*, and *UBLCP1*) sharing a conserved structural organisation in the catalytic core. Here again, highly conserved amino acids within this family of phosphatases bind metal-ions and phosphates necessary for phosphatase activity (Figure 1A) (Shi, 2009). HADs use an aspartate residue for nucleophilic attack, in contrast to the classical serine or cysteine residue (Shi, 2009). As such, HADs are unique in their enzymatic activity, explaining their lack of targeting by commonly used phosphatase inhibitors (Seifried et al., 2013). Structurally, the HAD catalytic core is arranged as a modified Rossmann fold, with additionally three structural signatures (cap, flap, and squiggle domain) that confer substrate specificity (Seifried et al., 2013). FCP1, encoded by *CTDP1*, contains several structural domains, such as an insertion in the catalytic core, a BRCA1-related C-terminal domain (BRCT), and a transcription factor interacting helix (TFIIF), important in substrate binding and thus phosphatase activity (Figure 1A).

## 3 Ser/Thr phosphatases in congenital diseases

The role of PSP phosphatases in inborn developmental disorders has only recently emerged, in part, thanks to major advancements in genetic technologies such as Next Generation Sequencing (NGS), and in part through emerging insights into the role of *de novo* mutations in genetic disease (Veltman and Brunner, 2012; de Ligt et al., 2013). These advancements lowered the cost of routinely screening for genetic alterations throughout the exome, and even through the whole genome, at base pair resolution, leading to a major expansion of novel congenital causes of disease. In particular, the application of so-called ‘trio sequencing’ methodology underscored the important role of germline *de novo* point mutations as major causes of rare sporadic monogenic diseases (Vissers et al., 2010; Gilissen et al., 2014).

Recently, many new initiatives have been taken to focus on deciphering until then unknown causes of disease. A prime example is the Deciphering Developmental Disorders (DDD) study in the United Kingdom, having screened over 33,000 parents and children as of January 2020, leading to the identification of 67 completely new disorders, including several involving phosphatase genes (Deciphering Developmental Disorders Study, 2015; Short et al., 2018; Wright et al., 2018; Kaplanis et al., 2020). This example highlights the explosion of newly identified genetic disorders during recent years, many of which linked to phosphatase dysfunction, and creating the need for more research into this subject.

In this review, we aim to create an overview of currently known, inborn genetic diseases caused by mutations in PSP phosphatase genes. Thus, we aim to provide a reference framework to further study these novel genetic disorders, and spur advancement in mechanistic insight in these diseases.

### 3.1 PPPs

#### 3.1.1 Protein Phosphatase 1

PP1 phosphatases form a myriad of holoenzyme complexes, with a diverse set of regulatory subunits or PIPs (Verbinnen et al., 2017). This way, PP1 can form more than 540 dimers, with an undefined number of trimers. Of the three catalytic subunit encoding genes, only *PPP1CB* has been defined as the cause of a congenital disease, *i.e*. Noonan-like syndrome with loose anagen hair-2. In addition, three major PP1 regulatory subunit genes also confer inborn disease upon mutation: *PPP1R12A, PPP1R15B,* and *PPP1R21*.

PP1 as a phosphatase is ubiquitously present in cells and tissues, and has many different functions, depending on localisation and expression of its regulatory subunit. In brain, expression of a constitutively active inhibitor of PP1, Inhibitor-1, improved learning and memory formation, while worsening recovery from ischemia-induced brain damage (Ferreira et al., 2019). Furthermore, in the striatum, PP1 is inhibited through DARPP-32 (dopamine and cAMP-regulated phosphoprotein of 32 kDa), a key player in dopamine signalling, when DARPP-32 is phosphorylated at Thr34 (Ferreira et al., 2019). Dysregulation of this inhibition is associated with several neurological and psychiatric diseases, such as Parkinson’s disease, drug addiction, and schizophrenia (Ferreira et al., 2019). The actin-binding PIPs, neurabin-1 and spinophilin, are required for neurite formation, target PP1 to post-synaptic densities, and regulate synaptic transmission and plasticity through regulation of AMPA- and NMDA-receptor dephosphorylation and signalling (Ferreira et al., 2019; Foley et al., 2021). PP1’s role in synaptic plasticity, in general, is to promote long-term depression and to inhibit long-term potentiation induction, and this is likely achieved by diverse PIPs (Foley et al., 2021). Inhibitor-2, for example, regulates PP1 in plasticity, not only by inhibiting PP1, but also by positively regulating PP1 function *in vivo* in memory formation (Foley et al., 2021). Further organ-specific functions of PP1 have been extensively described, such as in the heart (Klapproth et al., 2022), being involved in a wide variety of cellular pathways such as DNA-damage response, cell division and translation (Connor et al., 2001; Saurin, 2018; Holder et al., 2019; Campos and Clemente-Blanco, 2020), glycogen metabolism (Delibegovic et al., 2003; Kelsall et al., 2011), and embryonic development (Ferreira et al., 2019).

**FIGURE 2:**
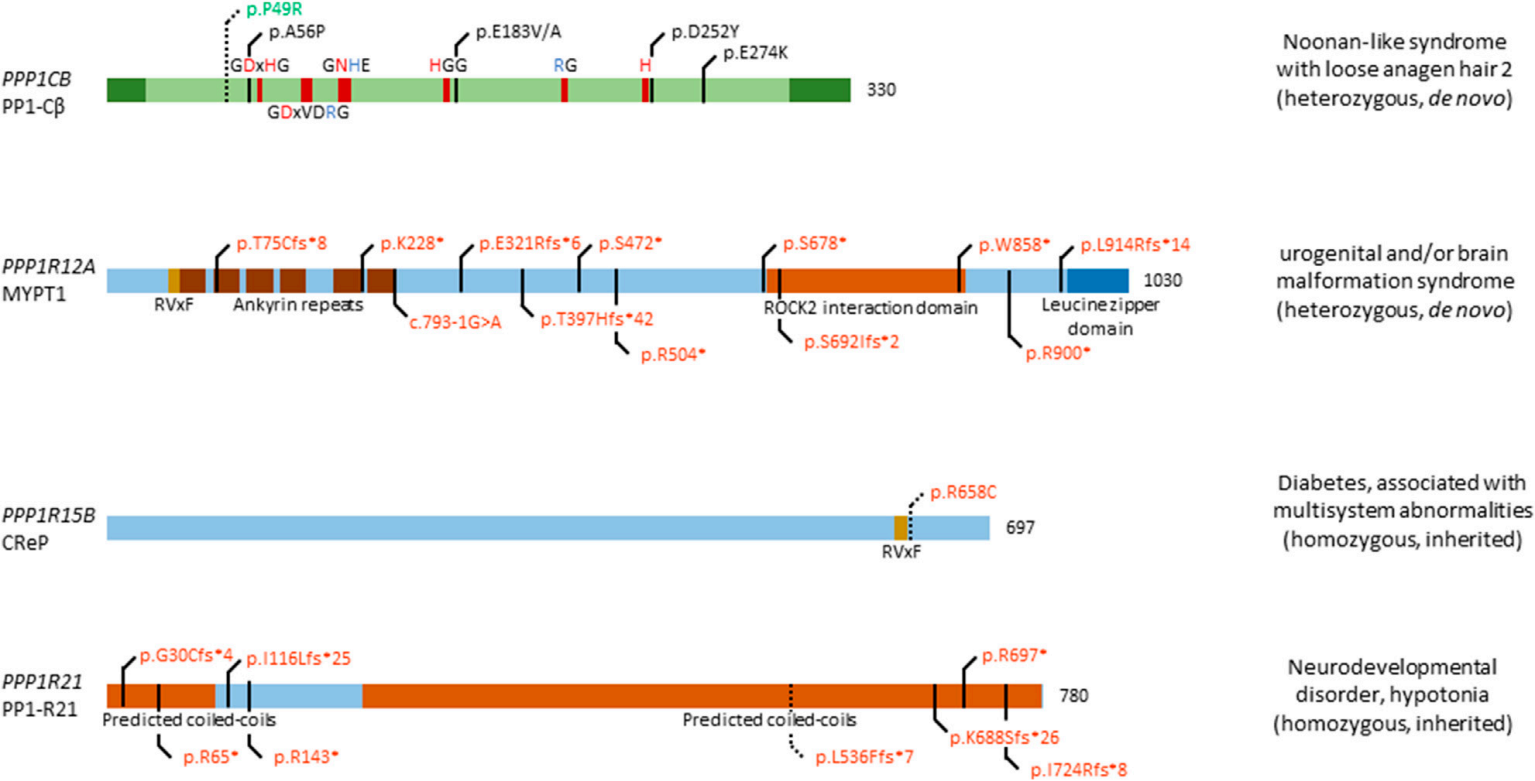
Overview of congenital disease-causing variants in subunits of PP1. In total, 5 distinct genetic variants have been found in *PPP1CB*, encoding the PP1 catalytic subunit β isoform; 12 variants in *PPP1R12A*, encoding the myosin targeting subunit protein 1 (MYPT1); 1 variant in *PPP1R15B*, encoding the Constitutive repressor of eIF2α phosphorylation (CReP); and 8 mutations in *PPP1R21*, encoding the PP1 regulatory subunit 21 (PP1-R21). Variants in green denote alleged or proven gain-of-function mutants, variants in red: loss-of-function mutants, variants in black: pathogenic mechanism unknown. Dotted lines near variants denote that functional data have proven pathogenicity. Additional structural and regulatory domains in the PP1 subunits are indicated, as well as conserved amino acids of the catalytic subunit (red amino acids denote metal binding, blue denote phosphate binding). RVxF denotes the most recurrent PP1 catalytic subunit binding motif.

The involvement of *PPP1CB* in Noonan syndrome-like disorders was first found in 2016, when four patients presented with typical symptoms of this disorder, including distinctive hair anomalies, developmental differences, and structural brain abnormalities (Gripp et al., 2016) (OMIM#617506). Typically, this disorder is caused by mutations in *SHOC2*, encoding a leucine-rich repeat scaffold protein, which in complex with PP1-Cβ and MRAS, activates the RAS/MAPK pathway (Motta et al., 2019). As such, it came as no surprise that mutations in *PPP1CB* lead to similar phenotypes. The four initially identified patients showed a diverse phenotype, with symptoms including developmental delay, hypotonia, macrocephaly, a prominent forehead, and cerebellar abnormalities, among others. In this study, two *de novo* heterozygous variants were found, a Pro49Arg substitution, and a Ala56Pro substitution (Gripp et al., 2016) (Figure 2). In the following years, a few more patients were identified, all carrying the heterozygous Pro49Arg substitution (Bertola et al., 2017; Zambrano et al., 2017; Maruwaka et al., 2022). Two years after initial reporting, Young *et al.* showed that mutations in *PPP1CB*, and other causative genes of this disorder, all promote formation of a ternary complex consisting of SHOC2*,* PP1-Cβ, and MRAS (Young et al., 2018). In this ternary complex, SHOC2 acts as a structural subunit, holding together the regulatory MRAS and PP1 Cβ subunit, leading to a very specific dephosphorylation of RAF kinases on an inhibitory site, and eventually, to overactivation of the MAPK pathway further downstream (Young et al., 2018). As such, gain-of-function (GOF) mutations in *PPP1CB* lead to Noonan syndrome-like disorders. Furthermore (other) mutations in *PPP1CB* have been implied as causative of intellectual disability and congenital heart disease. Ma *et al.* identified eight individuals with heterozygous, *de novo* variants in *PPP1CB*, presenting with intellectual disability, developmental delay, congenital heart disease, macrocephaly, short stature, and dysmorphic features (Ma et al., 2016). Five distinct variants were found, the Pro49Arg substitution, a Glu183 substitution to either a Val or Ala, a Asp252Tyr substitution, and a Glu274Lys substitution. Most of these mutations affect conserved regions of *PPP1CB*, indicating an important function of these specific amino acids (Figure 2). However, no further molecular studies were performed, and no connection to Noonan syndrome-like disorders was mentioned (Ma et al., 2016).

Among many PP1 regulatory subunits, we will highlight three genes found to be mutated in a specific congenital disease.

*PPP1R12A* encodes the PP1 Myosin phosphatase targeting subunit 1 (MYPT1), which forms a complex with PP1 catalytic subunit Cβ and M20/21 to form myosin phosphatase. This holoenzyme regulates function of non-muscle myosin II, and the interaction between myosin and actin in smooth muscle cells, through the phosphorylation state of the myosin light chain - with dephosphorylation of the light chain activating myosin function. MYPT1 is involved in embryogenesis by regulating cell adhesion and motility in the eye of *Drosophila*, embryonic elongation in *C. elegans*, gastrulation in *D. rerio*, and embryonic viability after 7.5 days post conception in mice (Kiss et al., 2019; Hughes et al., 2020). Furthermore, MYPT1 regulates cell proliferation and division through pRb (retinoblastoma tumour suppressor protein), merlin, and PLK1 (polo-like kinase 1), while acting as a tumour suppressor gene and modulating insulin signalling (Kiss et al., 2019). In the brain, MYPT1 accelerates the release of neurotransmitters and mediates the available pool of synaptic vesicles ready for release (Kiss et al., 2019). In development, MYPT1 has been shown to be of utmost importance through the modulation of the acto-myosin complex, which drives tissue morphogenesis (Kiss et al., 2019).

MYPT1 is found ubiquitously, with high expression in brain, thyroid gland, kidney and urinary tract, respiratory system, gastrointestinal tract, liver and gallbladder, and male and female tissues (Uhlén et al., 2015; Karlsson et al., 2021). Recently, twelve heterozygous *de novo* variants were found in twelve individuals (p.Thr75Cysfs*8, p.Lys228*, c.793-1G>A, p.Glu321Argfs*6, p.Thr397Hisfs*42, p.Ser472*, p.Arg504*, p.Ser678*, p.Ser692Ilefs*2, p.Trp858*, p.Arg900*, and p.Leu914Argfs*14), presenting with a neurological phenotype, urogenital malformations, or an overlap of both (Figure 2) (Hughes et al., 2020; Alkhunaizi and Chitayat, 2021) (OMIM#618820). Furthermore, a discordance between genotypic and phenotypic sex was observed in two individuals. The neurological phenotype includes, but is not limited to, brain malformations such as holoprosencephaly, macro- and microcephaly, dysmorphic facial features, seizures and a high frequency of developmental delay. Nine individuals presented with urogenital malformations, including malformations of the genitalia in both sexes, such as the presence of a uterus in XY individuals. No clear genotype-phenotype association could be established, given the wide diversity of phenotypic features, and the variants being spread among multiple exons and domains (Figure 2). However, all variants are likely loss-of-function due to premature stop codon gains, canonical splice acceptor site loss, or frameshifts followed by premature translation stops, in agreement with what authors concluded (Hughes et al., 2020), although no functional studies were performed to confirm this. The authors did nicely show expression of *PPP1R12A* in mouse embryonic brain tissue, and presence of MYPT1 in the developing lower urinary tract tissues (Hughes et al., 2020), consistent with its presumed role in embryogenesis of the brain and the genitourinary systems, and in sex development. Thus, further investigation of these twelve variants is warranted to conclusively prove loss-of-function, and to identify affected pathways caused by variations in *PPP1R12A.*

The ubiquitously expressed *PPP1R15B* gene, encoding the Constitutive Repressor of eIF2α Phosphorylation (CReP), is involved in protein kinase R-like endoplasmic reticulum kinase (PERK) signalling. PERK itself phosphorylates eukaryotic translation initiation factor 2α (eIF2α), thus downregulating protein translation (Abdulkarim et al., 2015). This signalling pathway is of importance in regulating stress on the endoplasmic reticulum, caused by an imbalance in unfolded protein load and the endoplasmic reticulum’s functional capacity. CReP continuously dephosphorylates eIF2α in basal conditions, thus allowing eIF2α to carry out its function as translation initiator. When the unfolded protein load becomes too large, PERK is activated, leading to phosphorylation and inhibition of eIF2α, thus lowering unfolded protein load in the endoplasmic reticulum (Abdulkarim et al., 2015). In 2015, two independent groups found a homozygous mutation in *PPP1R15B*, leading to a change of Arginine 658 to a Cysteine in four patients, of which two sets of siblings (Abdulkarim et al., 2015; Kernohan et al., 2015) (OMIM#616817). The Arg658Cys substitution is located in the conserved C-terminal functional core of the protein, near the RVxF-motif (Figure 2). This C-terminal core directs interaction between CReP and PP1 and mediates substrate specificity (Abdulkarim et al., 2015). All four patients present with neonatal-onset diabetes, short stature, microcephaly, and intellectual disability. Interestingly, homozygous loss-of-function mutations in *EIF2AK3*, encoding PERK, cause a similar phenotype, *i.e*. Wolcott-Rallison syndrome (OMIM#226980), characterized by neonatal diabetes associated with skeletal dysplasia and/or episodes of acute liver failure (Delépine et al., 2000; Biason-Lauber et al., 2002). This implies *PPP1R15B* mutations to be involved in PERK signalling and eIF2α dysphosphorylation. Accordingly, the p.Arg658Cys variant of CReP led to loss of PP1 catalytic subunit binding, in both patient-derived lymphoblasts and in a HEK293T overexpression model. As a consequence, increased constitutive phosphorylation of eIF2α was observed (Abdulkarim et al., 2015; Kernohan et al., 2015). Furthermore, it was shown that *PPP1R15B* was induced after ER stress in pancreatic β-cells in a PERK-dependent fashion, whereas silencing of *PPP1R15B* led to higher levels of eIF2α phosphorylation (Abdulkarim et al., 2015). Additionally, insulin secretion was impaired by *PPP1R15B* deficiency in β-cells, which then became sensitised to apoptosis (Abdulkarim et al., 2015). Lastly, RNA and protein levels of *PPP1R15B* were increased in patient-derived lymphoblasts, indicating a compensatory increase in expression to try to ameliorate the effects of CReP deficiency (Kernohan et al., 2015). Taken together, these data strongly implicate the p.Arg658Cys variant of CReP in altering PERK signalling and being deleterious to pancreatic β-cells (and likely other secretory tissues), resulting in long lasting, early-onset diabetes. As of yet, it remains unclear how *PPP1R15B* loss-of-function explains the short stature and brain-related phenotypes observed in affected patients.

Although *PPP1R21* is ubiquitously and highly expressed in most tissues (Uhlén et al., 2015; Karlsson et al., 2021), its function remains poorly understood. As a PP1 regulatory subunit, PP1-R21 plays a role in endosome formation, and co-localises with the early endosome marker EEA1. The same study showed expression of *Ppp1r21* in the developing cortical layers in murine brain, indicating a role in neurodevelopment (Rehman et al., 2019). Recently, several patients were identified to be carrying homozygous mutations in *PPP1R21*, all of them children of heterozygous carriers (Suleiman et al., 2018; Rehman et al*.*, 2019; Loddo et al*.*, 2020) (OMIM#619383). The first study identified three patients with homozygous null variants in *PPP1R21*, encompassing two homozygous nonsense variants (p.Arg697*, and p.Arg143*), and one homozygous frameshift variant (p.Gly30Cysfs*4) (Suleiman et al., 2018) (Figure 2). Another study, in the same year, identified four homozygous truncating variants, p.Ile116Lysfs*25, p.Ile724Argfs*8, p.Leu536Phefs*7, and p.Lys688Serfs*26 (Rehman et al., 2019). Most recently, a third study identified another homozygous stop variant, p.Arg65* (Loddo et al., 2020) (Figure 2). All patients shared a similar phenotype, with features including distinct facial dysmorphisms, intellectual disability, hypotonia, severe developmental delay, and brain abnormalities (Suleiman et al., 2018; Rehman et al., 2019; Loddo et al., 2020). One study performed deeper analysis of one variant, finding that expression of *PPP1R21* in patient-derived fibroblasts was lacking, and no protein could be observed, thus confirming that the variant was a null allele (Rehman et al., 2019). As PP1-R21 co-localises with an early endosome marker, the authors tested whether the uptake and clearance of transferrin-488 was affected in patient-derived fibroblasts and found that macro-autophagy is not disturbed by variants of *PPP1R21*, while clearance was mildly delayed. Furthermore, electron microscopy revealed a mild endo-lysosomal dysfunction, by nature of an increase in myelin figures in patient-derived fibroblasts (Rehman et al., 2019). Of note is that the authors only performed experiments with fibroblasts derived from one patient, carrying the p.Leu536Phefs*7 variant, which should be noted as a limitation of the study. Due to the poorly understood function of PP1-R21, no further molecular studies can be performed, such as dephosphorylation assays of a common substrate. Therefore, it is necessary to first understand the molecular and functional characteristics of PP1-R21, in order to understand the exact effect of variants in *PPP1R21* on cellular and brain function.

#### 3.1.2 Protein Phosphatase 2A

##### 3.1.2.1 PP2A-related neurodevelopmental disorders

PP2A phosphatases are holoenzymes, in most cases consisting of three distinct subunits: a scaffolding A-subunit, a catalytic C-subunit, and a regulatory B-subunit (Figure 1B). The genetic complexity of this phosphatase family in humans is high, as both the A- and C- subunits are encoded by two genes (*PPP2R1A, PPP2R1B,* and *PPP2CA* and *PPP2CB,* respectively), and the variable B-subunits, belonging to four different families, are encoded by 15 distinct genes: *PPP2R2A, PPP2R2B, PPP2R2C, PPP2R2D* (R2, B, PR55 or B55 family); *PPP2R3A, PPP2R3B, PPP2R3C* (R3, B″ or PR72 family); *PPP2R5A, PPP2R5B, PPP2R5C, PPP2R5D, PPP2R5E* (R5 or B′, PR61 or B56 family); and *PPP2R6A, PPP2R6B* and *PPP2R6C* (R6, B‷ or striatin family). This diversity in subunits results in more than 100 possible PP2A trimers, in which the B-subunit confers substrate specificity and localisation, the C-subunit catalytic activity, and the A-subunit structural integrity (Janssens and Goris, 2001; Reynhout and Janssens, 2019).

**FIGURE 3:**
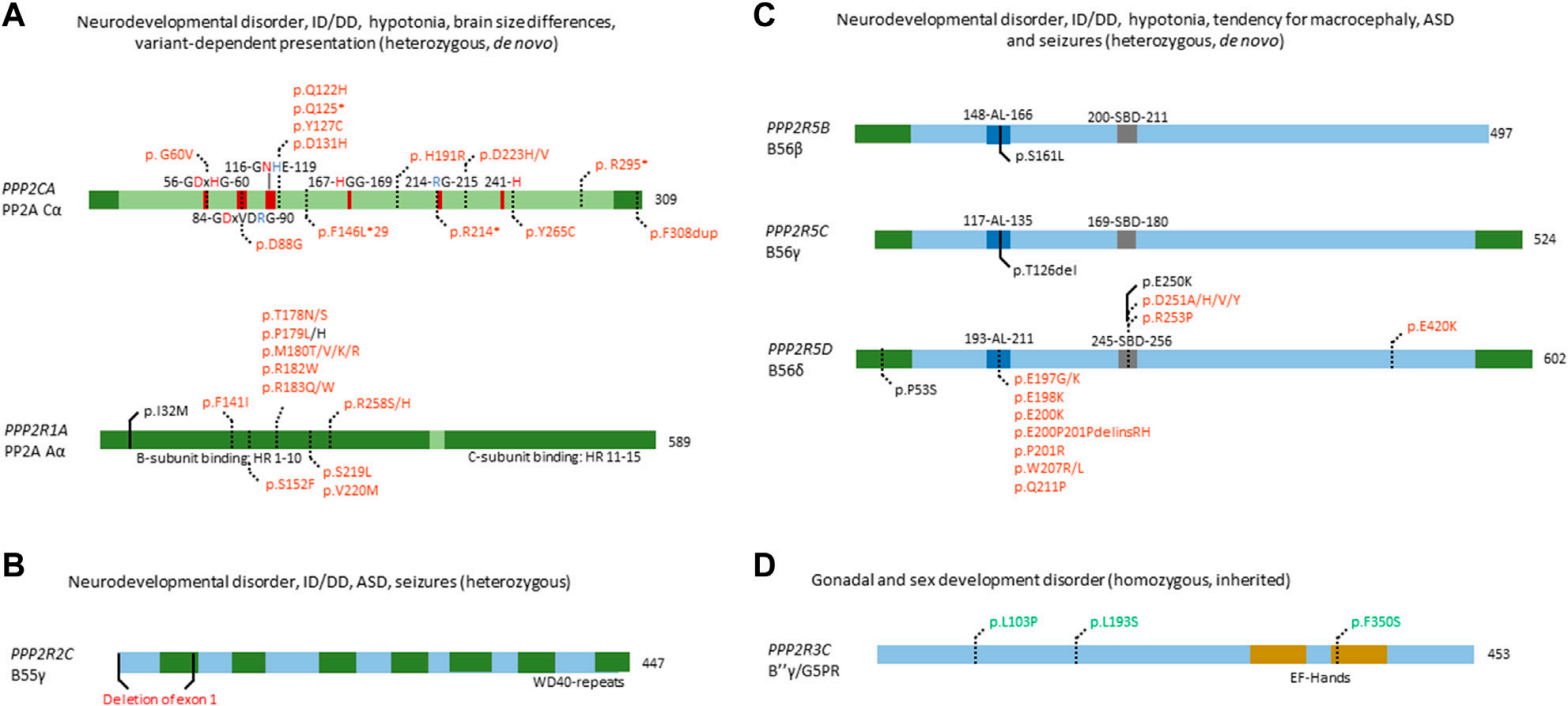
Overview of congenital disease-causing variants in subunits of PP2A. **(A**–**C)** Overview of variants in PP2A phosphatase genes related to neurodevelopmental delay and intellectual disability. **(A)** Mutations in *PPP2CA* and *PPP2R1A*, encoding the catalytic Cα and the structural Aα subunit, respectively, are indicated - as well as conserved residues in the catalytic subunit (red amino acids denote metal binding, blue denote phosphate binding), and the HEAT-repeats (HR) in the A-subunit that mediate binding to the C- and B-subunits. Variants in green denote alleged or proven gain-of-function mutants, variants in red: loss-of-function mutants, variants in black: pathogenic mechanism unknown. Dotted lines near variants denote that functional or molecular data have been reported. **(B)** Partial gene deletion of *PPP2R2C* (encoding B55γ regulatory subunit) causes an autosomal dominant intellectual disability syndrome. WD40 repeat: structural domain rich in Trp and Asp residues. **(C)** Mutations in *PPP2R5B* (encoding B56β), *PPP2R5C* (encoding B56γ) and *PPP2R5D* (encoding B56δ) all cause an ID/NDD disorder, often associated with macrocephaly. B56 subunit conserved domains and their residue numbering are indicated: AL, acidic loop; SBD, SLIM-binding domain. Variants in red denote proven loss-of-function mutants. Dotted lines near variants denote that molecular data were gathered, in most cases having proven pathogenicity. **(D)** PP2A phosphatase genes related to developmental abnormalities in non-neural tissues. Homozygous variants of *PPP2R3C*, encoding B″γ or G5PR, cause a gonadal and sex developmental syndrome. EF-hands: canonical Ca^2+^-binding motif. Variants in green denote alleged or proven gain-of-function mutants. Dotted lines near variants denote that functional data have proven pathogenicity.

Due to the omnipresence of PP2A in human cells, making up to 1% of the total cellular protein content (Remmerie and Janssens, 2019), PP2A is involved in a wide variety of cellular pathways and physiologic functions (Janssens and Goris, 2001; Reynhout and Janssens, 2019). Among the PP2A-regulated pathways are PI3K-Akt, mTOR, MAPK, Wnt, apoptosis, cell cycle control, and DNA damage response (Remmerie and Janssens, 2019). Given this wide variety of signalling pathways regulated by PP2A, it has been difficult to exactly delineate which pathway is under control of what regulatory subunit, as well as the exact effect of changes in PP2A function on signalling outcome. Thus far, most PP2A research has been performed in a cancer-context, where the large majority of PP2A trimers act as tumour suppressors (Meeusen and Janssens, 2018; Remmerie and Janssens, 2019). Since 2015, however, *de novo* mutations in several PP2A genes have been implicated as causative for neurodevelopmental or other inborn brain disorders (Houge et al., 2015; Loveday et al., 2015; Sandal et al., 2021; Verbinnen et al., 2021), while some inherited mutations in other PP2A genes mainly affected development of other organs (Guran et al., 2019). Specifically, PP2A-related neurodevelopmental disorders are characterized by mutations in *PPP2R1A* (Aα), *PPP2CA* (Cα), *PPP2R2C* (B55γ), *PPP2R5B* (B56β)*, PPP2R5C* (B56γ)*,* and *PPP2R5D* (B56δ), with most patients and mutations, so far, found in *PPP2R5D* (Figures 3A–C). Currently, more than 260 patients have been identified, while still more cases are being reported in literature, and more patients are being diagnosed every month (https://jordansguardianangels.org/). In contrast, mutations in *PPP2R3C* (encoding the regulatory B″γ or G5PR subunit) were found to be mainly associated with deficits in gonadal development (Guran et al., 2019) (Figure 3D), and mutations in the promotor of *PPP2R2B* (encoding B55β) with spinocerebellar ataxia (Holmes et al., 1999).

##### *PPP2CA* and *PPP2R1A*

PP2A A- and C-subunit expression is high in both developing and adult brain tissue, with a higher expression of *PPP2R1A* and *PPP2CA* as compared with their β isoform encoding genes, *PPP2R1B* and *PPP2CB* (Verbinnen et al., 2021). As such, PP2A has been shown to be important in brain development and function, with several mouse studies performed (Reynhout and Janssens, 2019; Verbinnen et al., 2021). Brain-specific PP2A Cα knockout in mice has been linked to microcephaly and cortical atrophy, while learning and memory was also impaired (Liu et al., 2018). Of note, a complete *Ppp2ca* or *Ppp2r1a* knockout in mice is embryonically lethal around E5.5-E6, due to impaired gastrulation and lack of mesoderm formation (Götz et al., 1998; Lange et al., 2017).

Fifteen heterozygous, *de novo* variants affecting *PPP2CA* have thus far been identified in sixteen individuals with a neurodevelopmental disorder, of which one full gene deletion, one insertion, four nonsense variants, and nine missense variants (Figure 3A) (Reynhout et al., 2019) (OMIM#618354). Patients presented with a heterogeneous set of symptoms, including mild to severe intellectual disability and developmental delay, autism spectrum disorder, seizures and brain abnormalities (Reynhout et al., 2019). Biochemical characterisation of the insertion, nonsense and missense variants revealed mainly losses-of-function, consistent with haploinsufficiency for milder cases, and dominant-negative action for more severe cases (Reynhout et al., 2019). In the latter, catalytic activity of the variant was completely abolished, but holoenzyme formation with specific B subunits, mainly belonging to the B56 family, was retained (Reynhout et al., 2019).

In *PPP2R1A*, 18 distinct heterozygous, *de novo* mutations have been found in 41 cases thus far (Figure 3A) (Houge et al., 2015; Wallace et al., 2019; Zhang et al., 2020; Lenaerts et al., 2021; Baker et al., 2022; Douzgou et al., 2022) (OMIM#616362). Here, patients can be classified into at least two clinical subsets, one milder and one more severe group. Both groups display typical PP2A-related neurodevelopmental symptoms such as hypotonia, intellectual disability and developmental delay. However, the mild subgroup of individuals tends to have macrocephaly, no epilepsy, and sometimes, microtia and loss of hearing, while patients in the severe subgroup tend to present with microcephaly, seizures, facial dysmorphisms, corpus callosum hypoplasia or agenesis, and sometimes, congenital heart defects (Lenaerts et al., 2021; Baker et al., 2022). Variants characterising the mild subgroup are located in HEAT-repeats 4 or 5 of the Aα subunit (Figure 3A), and show normal binding to B55 and B‴, but severely decreased binding to B56 subunits apart from B56δ (Lenaerts et al., 2021). In contrast, variants in the severe subgroup are located in HEAT-repeats 5, 6 or 7, with severe impairment of B55 binding and variable binding defects of B″ and B56, while B‴ and B56δ binding were retained (Lenaerts et al., 2021). One variant (p.Ser152Phe) did not show any PP2A subunit binding defects but caused a reduced number in spikes upon expression in primary mouse neurons, and was associated with a very mild phenotype (autism spectrum disorder but no intellectual disability) (Lenaerts et al., 2021). Thus, the wide variability of B-subunit binding defects could explain the diversity in clinical manifestations and could also confer different pathogenic mechanisms or even a combination of different mechanisms such as losses- and gains-of-function of specific PP2A holoenzymes (Verbinnen et al., 2021). Of note is the association between somatic *PPP2R1A* variants with several cancers, such as endometrial cancer and lung cancer (Remmerie and Janssens, 2019), and with some of these variants being identical to those found in the severely affected *PPP2R1A* subgroup of neurodevelopmental disorders. PP2A Aα is a well-established tumour suppressor, and mutations in *PPP2R1A* can contribute to tumorigenesis (Meeusen and Janssens, 2018; Remmerie and Janssens, 2019). Interestingly, most mutations are located in similar regions as intellectual disability-related mutations, disrupting binding of the A subunit to several B subunits (Haesen et al., 2016; Remmerie and Janssens, 2019). As such, it would be interesting to explore if intellectual disability-related mutations pose an increased cancer risk in patients, and whether lessons can be learned from both diseases to contribute to therapeutic options.

##### PPP2R2C

*PPP2R2C* has been implicated in causing autosomal dominant intellectual disability. This was established in a family with several affected individuals, presenting with intellectual disability, autism spectrum disorder, learning and behavioural problems, late-onset epilepsy, and premature foetal death (Backx et al., 2010). All affected individuals were heterozygous carriers of a translocation disrupting two genes, the extracellular matrix-encoding component *LAMA2* and *PPP2R2C,* encoding the PP2A B55γ subunit (Figure 3B). *LAMA2* is found to be recessively involved in congenital muscular dystrophy type-1A, with heterozygous carriers being unaffected. Thus, the authors ascribed the reported phenotype to the disruption of *PPP2R2C*. Interestingly, *PPP2R2C* is not expressed during embryonic development, but *Ppp2r2c* is expressed in all regions of the forebrain in adult mice, with highest expression in the hippocampus, which is involved in learning (Backx et al., 2010). No further functional studies were performed to elucidate the exact mechanism of disease, providing opportunity to further study the function of *PPP2R2C* in brain.

##### *PPP2R5D, PPP2R5C* and *PPP2R5B*

*PPP2R5D* is highly expressed during embryogenesis and in the adult brain, indicating its important role (Verbinnen et al., 2021). During embryogenesis, it plays a role in the cell cycle, being a negative regulator of CDC25 phosphatase by targeting PP2A to the nucleus (Biswas et al., 2020). Furthermore, B56δ plays a role in striatal dopaminergic neurotransmission, neurotrophic signalling, and tau phosphorylation (Biswas et al., 2020). However, *Ppp2r5d* knockout in mice showed no brain abnormalities or memory and learning defects, but a tauopathy instead (Louis et al., 2011). *Ppp2r5d* heterozygous gene trapping, however, showed weakened pre-pulse inhibition, correlating with glycogen synthase kinase-3β hyperphosphorylation (Kapfhamer et al., 2010). Interestingly, the double knockout of *Ppp2r5d* and *Ppp2r5c* proved embryonic lethal at day E12, despite single knockouts being viable (Dyson et al., 2022), perhaps indicating a functional overlap and rescue of one subunit by the other. In dopamine signalling, B56δ has been shown to regulate dephosphorylation of DARPP-32, an important dopamine regulator and inhibitor of PP1 (Ahn et al., 2007). Furthermore, B56δ plays an indirect role in the phosphorylation of tau, a major factor in Alzheimer’s disease (Louis et al., 2011; Yu et al., 2014). As such, *PPP2R5D* plays an important role in several aspects of brain function, in various molecular pathways.

Patients with *de novo* variants of *PPP2R5D* most commonly present with intellectual and developmental delay, hypotonia, macrocephaly, distinct facial features (*e.g.* frontal bossing) and less common symptoms, such as epilepsy, autism spectrum disorder, ADHD and early-onset Parkinsonism (Houge et al., 2015; Loveday et al., 2015; Shang et al., 2016; Yeung et al., 2017; Mirzaa et al., 2019; Kim et al., 2020; Hetzelt et al., 2021; Walker et al., 2021; Yan et al., 2021; Oyama et al., 2022) (OMIM#616355). Thus far, seventeen variants have been reported for *PPP2R5D* which are spread throughout the whole protein, but some seem to cluster around a conserved acidic loop, or a canonical short linear interaction motif-binding site (Figure 3C) (Verbinnen et al., 2021). Current literature states that these *de novo* variants act in a dominant-negative fashion, showing decrease in both A- and C-subunit binding, thus potentially trapping the substrate (Houge et al., 2015). Recently, however, it was found that different variants affect formation of the holoenzyme differently, and three distinct biochemical groups were identified: one where only C-binding was affected, one where only a common SLIM-containing interactor was affected, and one where both were affected (Oyama et al., 2022). This nicely correlated with the clinical findings, where severity of the phenotype was less when both binding partners were affected. In contrast, in the two subgroups where only one of the two binding partners were affected, the phenotypes were found to be more severe, consistent with a stronger dominant-negative effect (Oyama et al., 2022). However, downstream molecular effects of *PPP2R5D* mutations remain poorly understood, with one variant of B56δ (p.Glu420Lys) leading to a constitutive activation of the Akt-mTOR pathway when reconstituted in HEK293 cells (Papke et al., 2021). However, no other variants were tested, and the downstream molecular effects need to be further assessed to better understand the aetiology of *PPP2R5D-*related neurodevelopmental disorders.

Previous research has shown that *PPP2R5B* plays a role in neuronal development and function. For instance, B56β inhibits dendritic branching, through dephosphorylation of Akt in Akt-dependent dendritic branching (Brandt et al., 2008). However, gene knockouts of *Ppp2r5b* or *Ppp2r5c* were viable in mice, and showed no brain abnormalities (Verbinnen et al., 2021). Thus far, both *PPP2R5B* and *PPP2R5C* mutations have been found in one patient each, identified within a selected cohort of individuals exhibiting overgrowth (Loveday et al., 2015) (Figure 3C). Both these variants affect the same conserved acidic loop as mutated in *PPP2R5D*, which provides substrate recognition for B56β, and C subunit binding for B56γ (Cho and Xu, 2007; Saraf et al., 2010). Despite lack of biochemical characterisation, these variants are thought to act dominant-negative as well, mimicking the action of *PPP2R5D* variants. As such, further investigation of these variants is warranted to confirm this hypothesis. In addition, specific screening for these genes could increase the number of identified patients and potentially identify more variants.

Taken together, PP2A-related neurodevelopmental disorders are a diverse set of diseases, with multiple subunits of PP2A mutated, leading to a variety of symptoms. Despite this variety of affected subunits, remarkably similar phenotypes have been identified with common symptoms including intellectual disability, developmental delay, hypotonia, brain (size) abnormalities, and often, epilepsy and ASD. Despite being relatively well characterised molecularly, an exact cellular disease mechanism remains unknown, in terms of which neuronal substrates could be affected in their (de)phosphorylation. Thus, to fully understand these diseases, more research needs to be performed to pinpoint which molecular pathways are affected by variants in PP2A subunits. Due to the lowering costs of genomic sequencing tools, more patients are being identified worldwide, and additional variants are found that could help expand the current knowledge regarding molecular and biochemical properties of PP2A subunits.

##### 3.1.2.2 Other congenital PP2A-related diseases

Apart from PP2A-related intellectual disabilities, some other congenital mutations have been found in *PPP2R2B* (OMIM#604326) and *PPP2R3C* (OMIM#618419) (Figure 3D).

*PPP2R2B*, encoding the B55β subunit, was implicated as causative in spinocerebellar ataxia 12, with patients presenting with upper extremity tremor, progressing to head tremor, gait ataxia, dysmetria, dysdiadokinesis, hyperreflexia, paucity of movement, and abnormal eye movements - eventually developing dementia. Patients were found to be carrying a number of expanded CAG repeats, 133 nucleotides upstream of the transcription start site of *PPP2R2B* (Holmes et al., 1999). The authors concluded that this CAG expansion might affect *PPP2R2B* expression, and is probably causative due to the lack of such expansion in controls, and the correlation between expansion degree and disease (Holmes et al., 1999); however, no expression or functional studies were performed to conclusively point out *PPP2R2B* as the cause of spinocerebellar ataxia 12, or to elucidate the mechanism through which this disease manifests itself.

Homozygous *PPP2R3C* mutations were found to cause 46, XY gonadal dysgenesis, with patients presenting with female external genitalia despite an XY karyotype, a hypoplastic uterus, dysmorphic features, a common facial gestalt, myopathy and a thick muscular build, neuromotor delay, and intellectual disability (Guran et al., 2019) (OMIM#618419). *PPP2R3C*, encodes the Ca^2+^-regulated B″γ/G5PR subunit and is relatively ubiquitously expressed (Janssens et al., 2003; Zwaenepoel et al., 2008). The only studies on the role of *PPP2R3C* focussed on the immune system: knockout studies in mice have shown an important role of *Ppp2r3c* in B- and T-cells, regulating their development and proliferation (Reynhout and Janssens, 2019). However, until the identification of these patients, *PPP2R3C* was unknown to play a role in human sex development. Histology performed on digenetic gonads of patients revealed an absence of phospho-Sox9, a direct downstream target of PP2A indispensable for testicular development, thus indicating gain-of-function (Guran et al., 2019). Given the autosomal recessive inheritance of this disease, the authors also investigated the effect of heterozygous *PPP2R3C* variants on reproductive functions. They found that heterozygous fathers had teratozoospermia with severe head, acrosomal, and nuclear abnormalities. Heterozygous mothers reported oligomenorrhea and hypomenorrhea, or an unaffected menstrual cycle. Furthermore, B-lymphocyte viability was affected, with CD19^+^ B-lymphocytes from homozygous patients being more susceptible to apoptosis and proliferating less, compared to healthy controls (Guran et al., 2019). This study clearly links *PPP2R3C* mutations to gonadal dysgenesis, through a phospho-Sox9 deficiency, possibly by PP2A hyperactivity, but does not provide direct functional studies of the effect of these mutations on protein function and dephosphorylation. Thus, further functional studies, and possible other effects, apart from Sox9 dephosphorylation, are needed to fully characterise these variants.

#### 3.1.3 Calcineurin

Calcineurin (or PP2B) forms a complex of one catalytic subunit (encoded by three possible genes: *PPP3CA, PPP3CB* and *PPP3CC*), one Ca^2+^-binding regulatory subunit (*PPP3R1* or *PPP3R2*), and Ca^2+^/calmodulin to release auto-inhibition (Roy and Cyert, 2020) (Figure 1B). Like PP1 and PP2A, PP2B achieves substrate specificity through binding to SLIMs, such as the PxIxIT and LxVP motifs, that are present in their substrates, regulators and anchoring proteins (Wigington et al., 2020). Of the five human PP3 genes, one is implicated in genetic disease: *PPP3CA* (OMIM#618265, #617711) (Myers et al., 2017; Mizuguchi et al., 2018), while *PPP3CC* is linked to schizophrenia susceptibility (Gerber et al., 2003). Calcineurin is the most abundant calmodulin-binding protein in adult brain, mostly expressed in the neuronal cell body and processes (Khan et al., 2021). In the brain, calcineurin plays a key role in memory, synaptic plasticity, and synaptic transmission through the regulation of several pre- and postsynaptic targets, mainly ion channels and neurotransmitter receptors (Purkey and Dell'Acqua, 2020; Khan et al., 2021). Embryonic neural induction has been shown to be dependent on calcineurin, regulating the bone morphogenic protein-regulated Smad1/5 proteins after fibroblast growth factor stimulation (Cho et al., 2014). Congenital disease caused by *PPP3CA* alterations can be attributed to gain-of-function mutations, or loss-of-function mutations, evidenced by activation or repression of downstream calcineurin signalling, respectively. To note, all relevant mutations were found to be heterozygous, and most to be *de novo*.

**FIGURE 4:**
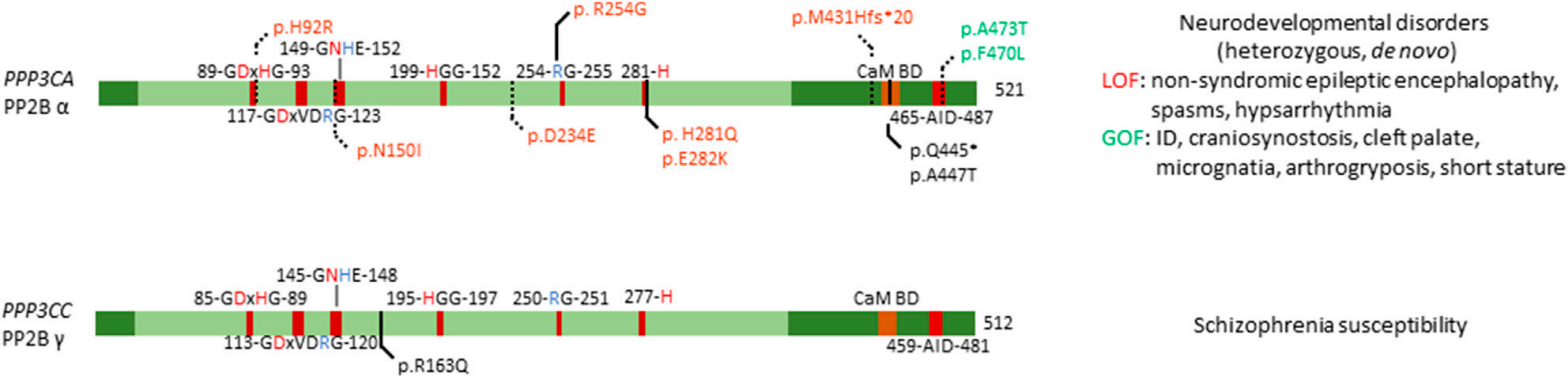
Overview of congenital disease-causing variants in calcineurin (PP2B, PP3) subunits. Overview of variants in the catalytic subunits of calcineurin, with conserved residues annotated. Red amino acids denote metal binding, blue denote phosphate binding. CaM BD, Ca^2+^-calmodulin binding domain; AID, auto-inhibitory domain. Variants in green denote alleged or proven gain-of-function mutants, variants in red: loss-of-function mutants, variants in black: pathogenic mechanism unknown. Dotted lines near variants denote that functional or molecular data have been obtained.

Mizuguchi *et al.* found several mutations, generally mapping to two distinct locations in the protein (Figure 4). One set of variants (p.His92Arg, p.Asn150Ile, and p.Asp234Glu) were found to affect the catalytic domain of PP2B α, leading to patients presenting with similar clinical features, such as non-syndromic epileptic encephalopathy with spasms and hypsarrhythmia (Mizuguchi et al., 2018). One other variant, p.Met431Hisfs*20 led to a similar phenotype, thus likely acting in a similar fashion as the previous mutations. These mutants in the catalytic domain were found to be loss-of-function, as evidenced by measuring calcium sensitivity in a yeast model system lacking *ppb1* (encoding a calcineurin catalytic subunit, CnA, in *S. pombe*)*,* making them intolerable to high calcium growth conditions. In this model, overexpression of *ppb1* with the conserved mutations in the catalytic domain failed to rescue this growth phenotype (Mizuguchi et al., 2018). Furthermore, two variants, p.Ala473Thr and p.Phe470Leu, were found to map to the auto-inhibitory domain of PP2B α, leading to different clinical features, such as intellectual disability, craniosynostosis, cleft palate, micrognatia, arthrogryposis and short stature (Mizuguchi et al., 2018). However, facial dysmorphism varied among patients. Using the same yeast model system, these mutations were shown to be gain-of-function, evidenced by their enhanced ability to rescue calcium intolerance as compared to wild-type *ppb1*. Additionally, both sets of mutants were tested for their ability to activate a downstream transcription factor Prz1, which confirmed the previously determined loss-of-function and gain-of-function mechanisms of these variants (Mizuguchi et al., 2018).

An earlier study identified six *PPP3CA* variants, p.His92Arg, p.Arg254Gly, p.His281Gln, p.Glu282Lys, p.Gln445*, and p.Ala447Thr, but no functional studies were performed (Figure 4) (Myers et al., 2017). Four of these substitutions affected the catalytic domain, i.e. His92Arg (as previously discussed), and Arg254Gly, His281Gln and Glu282Lys (Myers et al., 2017), while the other two variants seemed to reside close to the auto-inhibitory domain (Figure 4). All patients presented with profound to severe intellectual disability, developmental delay, and five out of six patients had epilepsy. These authors did not make a distinction between the two different sets of mutations, nor between phenotypes based on these mutations. As such, it would be interesting to re-evaluate the functional impact of these mutations using the same techniques Mizuguchi *et al.* had used, to see if their breakdown of the phenotype holds true in these patients as well. Furthermore, such assays could be used to further confirm their general conclusion that mutations in the catalytic domain led to a loss-of-function, while mutations in the auto-inhibitory domain led to gain-of-function, which explained the two separate phenotypes they noted in their 2018 study.

Several independent studies performed linkage analysis where they identified *PPP3CC* as a susceptibility locus for schizophrenia (Gerber et al., 2003). Further investigation showed that this susceptibility was conferred through a single point mutation, a substitution of amino acid 163, changing a positively charged arginine to a neutral glutamine (Figure 4) (Gerber et al., 2003). Furthermore, this study confirmed expression of *PPP3CC* in both adult and foetal brain tissue (Gerber et al., 2003). Despite strong genetic evidence, no causal relations have been found, and no functional studies have been performed to identify the exact mechanism of this susceptibility, or if neighbouring genes are the cause of this susceptibility.

### 3.2 PPMs

#### 3.2.1 PP2C family

Of the thirteen members of the PP2C family, four are linked to congenital disease: *PPM1B, PPM1D* (OMIM#617450)*, PPM1F,* and *PPM1K* (OMIM#615135)*. PPM1B* is one of the genes lost in the homozygous 2p21 deletion syndrome (Parvari et al., 2001, 2005; Jaeken et al., 2006), while mutations in *PPM1D* cause Jansen-de Vries syndrome (Jansen et al., 2017). Furthermore, *PPM1F* is a variant of unknown significance in familial intrahepatic cholestasis (Maddirevula et al., 2019), and *PPM1K* has a homozygous association with maple syrup urine disease (Oyarzabal et al., 2013).

**FIGURE 5:**
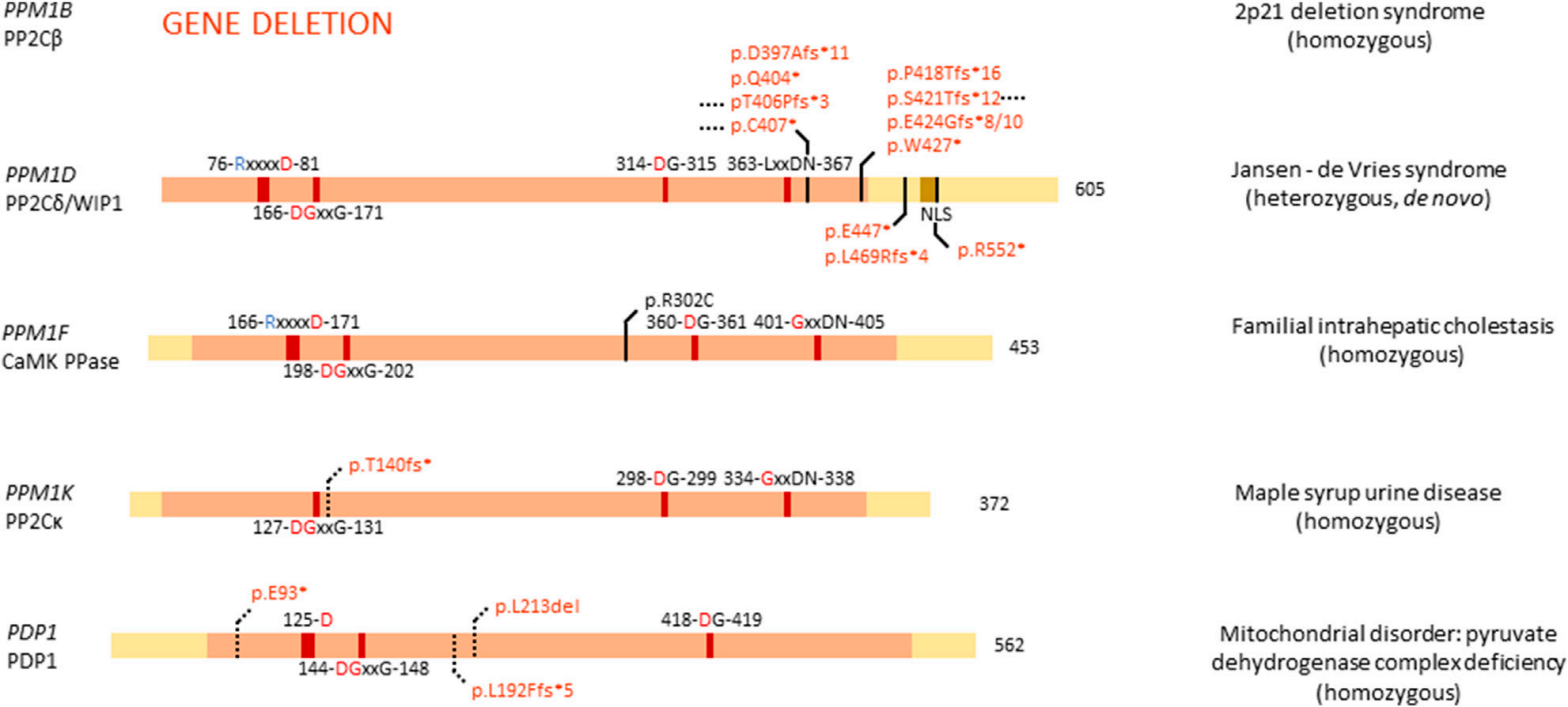
Overview of congenital disease-causing variants in PPM Ser/Thr phosphatases. Variants in the PPM phosphatases are displayed, with conserved amino acids and domains indicated. NLS, nuclear localisation signal. Red amino acids denote metal binding, blue denote phosphate binding. Variants in red denote alleged or proven loss-of-function mutants, variants in black: pathogenic mechanism unknown. Dotted lines near variants denote that functional or molecular data have been obtained.

PP2Cβ protein (encoded by *PPM1B*) is highly expressed in adult brain and gastrointestinal tissue, while *PPM1B* RNA is found in high quantities in muscle tissue (Uhlén et al., 2015; Karlsson et al., 2021). PP2Cβ is implicated in regulating necroptosis, senescence, adipogenesis, antiviral response, and the cell cycle (Kamada et al., 2020). Interestingly, *Ppm1b* deletion in mice has been reported to lead to embryonic lethality, with mRNA expression in wild-type mice being high at sperm maturation and in cell cycle arrested oocytes, while expression decreased between the two- and eight-cell stage of the embryo. Thus, PP2Cβ seems to play an important role in both early embryonic development and adult brain function (Kamada et al., 2020). Interestingly, *PPM1B* is fully lost in patients with homozygous 2p21 deletion syndrome, where 179,311 bp of DNA of chromosome 2p21 are deleted (Figure 5) (Parvari et al., 2001, 2005). Apart from *PPM1B*, three other genes are affected: the full type I cystinuria gene *SLC3A1*, the full, mostly uncharacterised, serine peptidase-encoding gene *PREPL*, and the first exon of the class I protein methyltransferase gene *C2orf34*. Patients present with cystinuria, neonatal seizures, hypotonia, severe somatic and developmental delay, facial dysmorphisms, and lactic acidemia. Researchers concluded that the cystinuria can be attributed to the heterozygous loss of *SLC3A1*; however, the exact role of the other affected genes has remained unclear in the development of the phenotype (Parvari et al., 2001, 2005). A further study of new families presenting with cystinuria-hypotonia found that in some patients only *SLC3A1* and *PREPL* were deleted, while *PPM1B* and *C2orf34* remained unaffected. Thus, researchers concluded that *SLC3A1* causes cystinuria, while *PREPL* likely causes the remaining phenotypic features. Consequently, the exact role of *PPM1B* in this disease remains unstudied, but is hypothesised to be minimal (Jaeken et al., 2006).

PP2Cδ or WIP1 (Wild-type p53 induced protein phosphatase 1), encoded by *PPM1D*, is a well-studied phosphatase, implicated in several processes, such as cell cycle regulation, metabolism, immune cell development and immune response, spermatogenesis, autophagy, and in cancer, it was established as an oncogene (Emelyanov and Bulavin, 2015; Kamada et al., 2020). *PPM1D* RNA is highly expressed in adult brain, while protein levels can be found in high quantities in brain, endocrine tissues, gastrointestinal tract, liver and gallbladder, kidney, male genitalia, and bone marrow and lymphoid tissues (Kamada et al., 2020). Furthermore, WIP1 is essential for adult neurogenesis through regulation of neural stem/progenitor cell differentiation. Researchers found that Wnt signalling is inhibited in aged neural stem/progenitor cells, barring them from differentiation, due to a lower expression of *PPM1D*. By stimulating Wnt signalling, neurogenesis was rescued in aged cells, and the authors proposed modulation of WIP1 can lead to the same effect (Zhu et al., 2014).

Given the high expression and important role of *PPM1D* in brain tissue, it came as no surprise that mutations in *PPM1D* can cause disease related to brain development. *De novo*, heterozygous mutations in *PPM1D* have indeed been identified as the cause of Jansen–de Vries syndrome, with patients presenting with mild to severe intellectual disability and/or developmental delay, autism spectrum disorder, ADHD, anxiety disorders, hypotonia, broad-based gait, facial dysmorphisms, and periods of fever and vomiting (Jansen et al., 2017). All identified mutations in *PPM1D* appeared nonsense or frameshift mutations, and are located in the last or next-to-last exon, thus leading to an escape of nonsense-mediated decay and normal expression of the protein (Figure 5). However, all variants of *PPM1D* lack the nuclear localisation signal (NLS), probably barring the protein from entering the nucleus, while the wild-type protein still does. WIP1 plays an important role in cellular stress, notably in the p53 pathway following DNA damage (Fiscella et al., 1997), where it regulates the phosphorylation state of CHK1 (Checkpoint kinase 1) and p53 itself (Lu et al., 2005). Furthermore, WIP1 regulates p38 MAPK signalling, creating a feedback loop with p53, since *PPM1D* is induced by p53 (Choi et al., 2002). Thus, it is of no surprise that somatic *PPM1D* gain-of-function mutations have also been implicated in cancer, including breast and colon cancer (Emelyanov and Bulavin, 2015). Interestingly, in Jansen–de Vries syndrome, the cellular stress pathway induced by ionizing radiation seems unaffected by mutations in *PPM1D*, possibly due to the presence of the wild-type allele. However, cells derived from patients showed a growth disadvantage when compared to control cells (Jansen et al., 2017). Furthermore, *PPM1D* was found to be widely expressed in foetal organs, such as liver, skeletal tissue, and brain, suggesting an important role in foetal development. Large database queries showed that *PPM1D* is under constraint for missense mutations, while it seems tolerant for loss-of-function mutations. Thus, it is hypothesised that pathophysiological mechanisms underlying ID-associated *PPM1D* mutations are complex in origin, with a C-terminally truncated protein probably being more disruptive than full loss of the protein, in line with a dominant-negative disease mechanism (Jansen et al., 2017). Here as well, no functional phosphatase activity assays were performed, which could shed more light on the exact mechanism through which C-terminally truncated WIP1 variants causes Jansen–de Vries syndrome.

*PPM1F* is ubiquitously expressed in human tissue and has been shown to play a role in regulating the kinesin motor complex, mDia1- and RhoA-dependent transcription mediated by serum response factor, neurofilament L function, oxidative stress response, depression, anxiety, cancer, Alzheimer’s disease, diabetes and inactivation of Ca^2+^/calmodulin-dependent protein kinase II (CAMKII) - hence its frequently used protein name, CAMK phosphatase (Kamada et al., 2020). Furthermore, *PPM1F* has been implicated to play a role in familial intrahepatic cholestasis, where one affected, consanguineous, family was identified (Maddirevula et al., 2019). One sibling presented with fatal liver failure at age 13, while another sibling presented with neonatal low gamma-GT cholestasis and jaundice, and indications of sclerosing cholangitis at age 19. At age 21, the patient suffered from short stature, portal hypertension, hypothyroidism, and a hyperpigmented tongue. This family was identified to carry a homozygous *PPM1F* variant, p.Arg302Cys (Figure 5). Interestingly, in the same study the authors identified a family with similar symptoms, but with mutations in a different gene, *KIF12*. As both *KIF12* and *PPM1F* play a role in centrosome orientation and kinesin-mediated transport, the authors hypothesised this disease as a cholangociliopathy caused by primary cilia dysfunction (Maddirevula et al., 2019). Important to note is that *PPM1F* mutations have only been found in one family, and no functional studies have been performed on the exact effect of this mutation on phosphatase binding and function.

Expression of *PPM1K*, encoding PP2Cκ, is highest in heart and brain but is otherwise ubiquitous (Uhlén et al., 2015; Karlsson et al., 2021). Within cells, PP2Cκ localises to the mitochondria where it regulates mitochondrial permeability, making it indispensable for cell survival and development, and cardiac function (Kamada et al., 2020). Furthermore, PP2Cκ regulates the degradation of the branched-chain α-keto acid dehydrogenase complex, and the stemness of hematopoietic stem cells (Kamada et al., 2020). As the sole regulator of the branched-chain α-keto acid dehydrogenase complex, PP2Cκ has been implicated in a mild variant phenotype of maple syrup urine disease. A homozygous deletion of base pairs 217T and 218A in the *PPM1K* cDNA has indeed been found to be causally linked to this disease. This 2-bp-deletion creates a C-terminally truncated protein through a frameshift, leading to a premature stop codon after Thr140 (Oyarzabal et al., 2013) (Figure 5). Maple syrup urine disease is caused by accumulation of valine, leucine, isoleucine, and their respective branched-chain α-keto acids, in cells and body fluids through a deficient activity of the branched-chain α-keto acid dehydrogenase. This accumulation leads to a range of mild to severe clinical phenotypes, including ketoacidosis, ataxia and neurological symptoms, due to the accumulation of these neurotoxic metabolites. *PPM1K* is the only known phosphatase regulator of the phosphorylation state of branched-chain α-keto acid dehydrogenase, where association between PP2Cκ with branched-chain α-keto acid dehydrogenase leads to activation of the complex (Zhou et al., 2012). Researchers performed functional studies in fibroblasts derived from patients, which contain the p.Thr140fs* variant, and found a decrease in function of branched-chain α-keto acid dehydrogenase, which was rescued by expression of wild-type *PPM1K* (Oyarzabal et al., 2013). Additionally, reactive oxygen species were found in higher concentrations in cells lacking functional *PPM1K*, and JNK and p38 kinases were induced. However, no change was observed in cell cycle progression. These experiments provided conclusive proof that a loss-of-function variant in *PPM1K* causes a mild phenotype of maple syrup urine disease and is a prime example of the direct effect of genetic variation in a Ser/Thr phosphatase gene on normal cell function (Oyarzabal et al., 2013).

#### 3.2.2 PHLPP and PDP

Of the four remaining PPM phosphatases, *PHLPP1*, *PHLPP2*, *PDP1,* and *PDP2*, only *PDP1* is thus far implicated in congenital disease (OMIM#608782). As one of two possible catalytic subunits of pyruvate dehydrogenase phosphatase, *PDP1* is predominantly expressed in skeletal muscle, while *PDP2* is more abundant in liver. PDP1 localises to the mitochondrial matrix, where it catalyses the dephosphorylation of pyruvate dehydrogenase, thus activating it (Lawson et al., 1993; Maj et al., 2006). Variants in *PDP1* can cause pyruvate dehydrogenase complex deficiency, a heterogeneous mitochondrial disorder with varying phenotypes, such as fatal infantile lactic acidosis, chronic neurological dysfunction, and intermittent ataxia (Maj et al., 2006; Cameron et al., 2009; Bedoyan et al., 2019). So far, three homozygous mutations that cause pyruvate dehydrogenase complex deficiency have been identified in *PDP1*: a 3-bp-deletion leading to deletion of leucine 213, a G-T transversion leading to a premature stop codon at amino acid 93, and a 1-bp-duplication at base pair 575, leading to a premature stop codon at amino acid 192 (Figure 5). Of all mutants, patient-derived fibroblasts were cultured, and activity of pyruvate dehydrogenase complexes measured, showing a decrease in activity of all three variants (Maj et al., 2006; Cameron et al., 2009; Bedoyan et al., 2019). This led to the conclusion that variants in *PDP1* are loss-of-function and cause a decrease in pyruvate dehydrogenase complex activity, thus contributing to disease. To note is that none of the three studies performed specific phosphatase activity assays of the variants to fully elucidate the exact molecular effect of these variants on PDP1 action.

### 3.3 HADs

**FIGURE 6:**
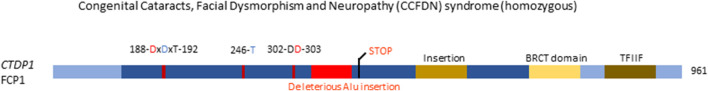
Congenital disease-causing variants in the HAD Ser/Thr phosphatases. Variants in the HAD phosphatase FCP1, encoded by *CTDP1*. Domains and conserved amino acids are indicated. Red amino acids denote metal binding, blue denote phosphate binding. Variants in red denote alleged or proven loss-of-function mutant. Dotted lines near variants denote that functional or molecular data have been obtained.

Of the seven genes encoding HAD monomeric phosphatases (*CTDP1, CTDSP1*, *CTDSP2*, *CTDSPL*, *CTDSPL2*, *CTDNEP1*, and *UBLCP1*) only the first one is currently known to cause an inborn genetic disease (OMIM#604168) (Varon et al., 2003). *CTDP1* encodes FCP1, a phosphatase that dephosphorylates serine 2 and serine 5 in the Tyr-Ser-Pro-Thr-Ser-Pro-Ser repeats at the C-terminal domain of RNA polymerase II, thus activating transcription initiation (Seifried et al., 2013), and is relatively ubiquitously expressed (Uhlén et al., 2015; Karlsson et al., 2021). A single point mutation in *CTDP1* was identified as the cause of a diverse phenotype in the Vlax Roma population, including congenital cataracts, facial dysmorphisms, and neuropathy, referred to as CCFDN (Congenital Cataracts, Facial Dysmorphism, and Neuropathy) syndrome (Varon et al., 2003). CCFDN syndrome is caused by a single, homozygous point mutation in an intronic, antisense Alu element between exons 6 and 7 of *CTDP1*, substituting a C for a T. This substitution leads to the creation of a donor splice site 389 bp downstream the end of exon 6 of *CTDP1* (Figure 6). Thus, the mutation creates the opportunity for alternative, aberrant splicing, where 95 nucleotides belonging to the antisense Alu sequence are inserted in the processed *CTDP1* mRNA. Due to this insertion, a frame shift of the reading frame occurs, leading to a premature termination signal, 17 codons downstream of the end of exon 6. Following this premature termination signal, it is hypothesised that the subsequent variant of FCP1 will be either non-functional or undergo nonsense-mediated decay (Varon et al., 2003). Interestingly, the wild-type transcript was still present in the affected patient, showing that both normal and aberrant splicing occur, causing a partial deficiency. Further investigation is warranted to conclusively confirm if the disease-causing variant of FCP1 indeed does undergo nonsense-mediated decay, or if, when not decayed, it loses functionality.

## 4 Conclusions and future perspectives

Given the lowering costs of exome and genome sequencing, it should be of no surprise that more genetic diseases have been identified during the last decade than at any time before. In particular, the notion that a small number of *de novo* mutations occurring during gametogenesis or post-zygotically, can become a major cause of developmental disorders (Veltman and Brunner, 2012; de Ligt et al., 2013), has not only resulted in providing final, sometimes long-sought after, diagnoses to many patients and families, but has also provided novel research opportunities to better understand the normal and diseased function of proteins.

Until recently, and unlike protein kinases, mutations in serine/threonine protein phosphatases (PSP) were not commonly associated with disorders of human development. Overall, of 38 human genes encoding PSP catalytic subunits or monomeric phosphatases, 10 were found to be affected in congenital disorders or were associated with an increased disease predisposition: *PPP1CB*, *PPP2CA*, *PPP3CA*, *PPP3CC*, *PPM1B*, *PPM1D*, *PPM1*F, *PPM1K*, *PDP1* and *CTDP1,* thus representing members of all three PSP subfamilies. In addition, 9 genes encoding a regulatory subunit of one of the multimeric PSP phosphatases were also among the congenital disease genes: *PPP1R12A*, *PPP1R15B* and *PPP1R21*, encoding regulatory subunits of PP1, and *PPP2R1A*, *PPP2R2C*, *PPP2R5B*, *PPP2R5C*, *PPP2R5D* and *PPP2R3C*, encoding subunits of PP2A.

In most cases, PSP gene mutations caused losses-of-function of the associated gene products, with just a few examples of gain-of-function variants (in *PPP1CB*, *PPP3CA* (some) and *PPP2R3C*). This observation has important ramifications for potential therapeutic interventions, as in general, it is more difficult to restore damaged phosphatase activity or expression by pharmacologic modulators, than to inhibit uncontrolled phosphatase activity or to interfere with increased phosphatase-substrate binding. In the former cases, alternative targeting of the relevant PSP downstream targets, *e.g*. by interfering with the upstream protein kinase activities, remains an appealing option to compensate for the loss of PSP activities. However, this necessitates knowledge about the affected physiologic substrates of the phosphatase, dysphosphorylation of which is causally involved in certain disease symptoms, as well as knowledge about the identity of the upstream kinase(s), and these insights are still often lacking. On the other hand, several PSP phosphatase activating pharmaca or peptide mimics did recently emerge (Wang et al., 2010; De Munter et al., 2013; Haslbeck et al., 2015; Brautigan et al., 2021), although their precise mechanisms-of-action often remain unclear or are under debate (Vit et al., 2022), and it seems unlikely that these compounds will be able to restore the function of genetically inactivated phosphatase gene products. In contrast, it has been known for a long time that potent pharmacologic PPP inhibition can be achieved by several naturally occurring toxins, such as calyculin A or microcystin-LR (Swingle et al., 2007; Zhang et al., 2013), while many PPM and HAD phosphatase inhibitors were identified through chemical compound library screening (Rogers et al., 2006; Sierecki et al., 2010; Zhang et al., 2013; Pecháčková et al., 2017). However, all these inhibitors target the highly conserved catalytic site of PSP phosphatases and are thus not very selective. Hence, it would be interesting to further explore the possibilities of finding pharmacological inhibitors that specifically target PPP holoenzymes, the non-catalytic domains of monomeric PSP phosphatases, or PSP enzyme-substrate complexes (Gilmartin et al., 2014; Krzyzosiak et al., 2018) to increase their specificity and avoid the undesirable pleiotropic actions (side effects) of catalytic site-targeted PSP inhibitors. Alternatively, novel gene therapy approaches, using improved CRISPR or RNA interference technology, and aiming to selectively edit or downregulate the mutation at DNA or mRNA level, remain valuable surrogates to pharmacologic interventions.

In general, the functional impact of identified mutations is easier to understand and to investigate in the monomeric PSP phosphatases, while this can be much more complex in the multimeric phosphatases, especially if mutations affect the common catalytic C-subunit (or, as is the case for PP2A, the common scaffolding A-subunit), and thus may have different effects on different holoenzyme complexes. For example, extensive molecular characterisation of *PPP2CA* and *PPP2R1A* variants has revealed an unexpectedly high complexity of holoenzyme assembly defects that are still incompletely understood, especially at the functional level, but that certainly could explain the opposite clinical features observed in some affected cases (*e.g.,* macro- vs. microcephaly). Moreover, it brings up the provocative question how different variants within a single affected PP2A gene, and variants in different PP2A genes may, or may not, relate to each other and affect similar pathways, potentially resulting in common, or different, molecular and/or clinical subgroups. The latter may have, again, important consequences for therapeutic intervention strategies.

Remarkably, in this overview of PSP congenital disorders, we often have encountered phenotypes that are specifically associated with brain dysfunction or dysmorphology, despite mutations being omnipresent in the human body, and the widespread tissue expression of most PSP genes. Thus, we must conclude that PSP-dependent protein dephosphorylation has a major potential for regulation of brain development and function. Nevertheless, some PSP disorders manifested themselves in other organs too, including the (uro)genital and endocrine systems, and were, in these cases, often linked to alterations in specific molecular pathways.

Thus far, few biochemical and molecular studies have been performed to investigate the effect of mutations on PSP protein function, downstream targets and associated pathways. Probably, the best-studied diseases in these respects are those involving mutations in *PPP1CB*, *PPP1R15B*, *PPM1K* and *PDP1*, followed by the *PPP2*- and *PPP3CA*-related neurodevelopmental disorders. Even in these relatively better understood diseases, knowledge is still incomplete, and in particular, the further identification of downstream targets and effectors could elucidate the exact mechanisms through which these diseases act, and possibly open up therapeutic avenues. To this end, new emerging technologies to identify PSP phosphatase substrates have been proposed that could be of value (Fahs et al., 2016), but there is still a long way to go, as the complexity is major. Of primary importance to achieve these goals, seems the generation and comprehensive characterisation of appropriate *in vitro* and *in vivo* disease models, such as appropriate knockout or knockin mice, and patient-derived cell models, including induced pluripotent stem cells that can be re-differentiated into the appropriate cell types. Finally, the number of diagnoses and of new variants affecting PSP genes is likely still to increase further in the near future, which may also help to identify new PSP functional domains and regulation mechanisms that could be of relevance to help decipher their physiologic functions.

In conclusion, PSP genes are obviously new players in congenital developmental disorders, mainly affecting the brain. On the longer term, this opens up new opportunities for clinical intervention, although much more research is required on the shorter term to understand through which mechanism(s) these gene variants cause the associated clinical phenotypes.
